# Effects of Dietary Supplementation with Extruded Linseed on Growth Performance and Meat Quality of Young Holstein Bulls

**DOI:** 10.3390/ani15142123

**Published:** 2025-07-17

**Authors:** Stella Dokou, Maria Eleni Filippitzi, Anestis Tsitsos, Vasiliki Papanikolopoulou, Stergios Priskas, Vangelis Economou, Eleftherios Bonos, Ilias Giannenas, Georgios Arsenos

**Affiliations:** 1 Laboratory of Animal Nutrition, School of Veterinary Medicine, Faculty of Health Sciences, Aristotle University of Thessaloniki, 54124 Thessaloniki, Greece; igiannenas@vet.auth.gr; 2 Laboratory of Animal Production Economics, School of Veterinary Medicine, Faculty of Health Sciences, Aristotle University of Thessaloniki, 54124 Thessaloniki, Greece; mefilippi@vet.auth.gr; 3 Laboratory of Animal Food Products Hygiene–Veterinary Public Health, School of Veterinary Medicine, Faculty of Health Sciences, Aristotle University of Thessaloniki, 54124 Thessaloniki, Greece; tsitanes@vet.auth.gr (A.T.); boikonom@vet.auth.gr (V.E.); 4 Laboratory of Animal Production and Environmental Protection, School of Veterinary Medicine, Faculty of Health Sciences, Aristotle University of Thessaloniki, 54124 Thessaloniki, Greece; vipapani@vet.auth.gr (V.P.); stpriskas@vet.auth.gr (S.P.); arsenosg@vet.auth.gr (G.A.); 5 Laboratory of Animal Production, Nutrition and Biotechnology, Department of Agriculture, University of Ioannina, 47132 Arta, Greece; ebonos@uoi.gr

**Keywords:** extruded linseed, Holstein bulls, meat quality, fatty acid profile

## Abstract

Beef production in Greece is a sector that has been characterized by a decline in both the output and the number of beef-producing animals over the last decades. The major challenge is low beef self-sufficiency; only 19.1% of demand is met by domestic production. The latter leads to a growing reliance on imports of both live animals and carcasses. Hence, the fattening of young bulls from dairy breeds could be an option to address this challenge subject to improving the quality of produced meat. The objective of the present study was to investigate the effects of extruded linseed in the diet of young bulls on their performance and meat quality. Sixty-eight young Holstein bulls were equally assigned in two experimental groups: the control group (CON, *n* = 34) and Linseed Group (LS, *n* = 34). Bulls in the CON group received a basal total mixed ration while LS young bulls were offered the same basal ration supplemented with linseed (5% on dry matter basis) during the final fattening stage. All bulls were subjected to three individual weightings at the beginning, the middle and the end of the trial. The feed offered was recorded daily and feed refusals were weighed for each pen to calculate feed intake. After slaughter, the *Longissimus dorsi* muscle from each carcass was collected to evaluate meat pH, color, chemical composition, tenderness and fatty acid profile. Analysis of variance was used to evaluate the effect of dietary intervention on performance and examined meat parameters, with significance set at *p* < 0.05, using SPSS software (version 29.0). Average daily gain, dry matter intake and feed conversion ratio were not affected by the dietary intervention (*p* > 0.05). Similarly, carcass yield and dressing percentage remained unaffected (*p* > 0.05). Adding extruded linseed did not result in differences in meat quality traits (*p* > 0.05), except for meat pH, which was significantly decreased in the LS group (*p* < 0.05), indicating more efficient post-mortem glycolysis. Finally, the inclusion of extruded linseed resulted in higher levels of α-linolenic acid in the meat (*p* < 0.05). These results suggest that including 5% extruded linseed (on a DM basis) in the diet of young Holstein bulls increased meat n-3 content, improved beef pH and maintained production performance.

## Simple Summary

Beef production in Greece is characterized by low self-sufficiency, with domestic output covering only 19.1% of national needs. As a result, the country relies on imports of live cattle and beef carcasses to meet demand. Utilizing young bulls from dairy breeds may serve as a useful strategy to support local production, especially if their meat quality is improved. Hence, this study aimed to evaluate the effects of extruded linseed dietary supplementation on Holstein bulls’ performance and overall meat quality. Sixty-eight bulls were allocated into two groups: one received a finishing diet and the other received the same diet with 5% extruded linseed added. Growth performance parameters were monitored, and meat samples were subjected to physicochemical analysis. The results showed that extruded linseed did not affect growth performance and most meat quality traits. However, it improved meat pH and increased meat n-3 content. Overall, this study suggests that feeding Holstein bulls extruded linseed could improve the nutritional value of beef without affecting animal performance.

## 1. Introduction

Beef cattle production in Greece is a sector characterized by deficiency, primarily stemming from an inadequate population of producing animals. This limitation has intensified over the last decades [1]. As a result, imports of live animals and carcasses, mainly from other European countries, are a necessity to meet the domestic demand. The mitigation of this deficiency could be achieved by expanding the national beef cattle herd or by channeling cattle from other production sectors into beef production. The latter approach could be implemented by utilizing young bulls, originating from the surplus calf population of dairy cattle operations. Currently, in Greece, approximately 90,000 dairy cattle are reared, predominantly of the Holstein breed [2]. Young bulls of these operations could help reduce Greece’s long-standing production deficit while supporting sustainability and local economies [3]. Although there are no official data documenting the exact fate of young Holstein bulls in Greece, these animals are widely considered as by-products of the dairy industry and are often not exploited to their full potential for beef production. This situation is not unique in Greece and has also been reported in other countries [4,5,6]. Notably, countries such as the United States, New Zealand and Ireland, have successfully integrated young Holstein bulls into their beef supply chains [4,5,6].

The Holstein breed is primarily selected for milk production. This genetic selection has come on expense of meat production potential, particularly in terms of dressing percentage and carcass conformation [7]. Consequently, the beef industry often considers these animals as a “by-product” of the dairy sector, inferior to purebred beef cattle. These issues translate to limitations for farmers, who are offered lower market prices for the meat of these animals [6]. Considering these challenges, efforts should focus on meeting market requirements and enhancing consumers’ appeal for meat derived from these animals.

Toward these efforts a plethora of studies have investigated methods for improving meat production potential and the quality of young Holstein bulls. These methods were either non-dietary, such as male castration [8], or dietary interventions, including the use of natural feed additives [9] and fats supplementation [10]. Additionally, beef health perception is of increasing importance for modern consumers. Hence, beef research has prioritized health-conscious approaches, such as enhancing beef PUFA and omega-3 content, through dietary interventions [11]. In this context, linseed is proposed as a feed ingredient due to its high nutritional value and unique fatty acid (FA) profile, which is particularly rich in omega-3 FAs. As a result, it represents an attractive dietary option for promoting animal growth and enhancing the quality of the final product [12].

Various studies have investigated the effects of linseed supplementation on production performance and the meat quality of purebred beef cattle [13,14] and lambs [15,16], reporting increased dressing percentage and a healthier meat FA profile, characterized by higher levels of n-3 FAs. However, the functional efficacy of linseed is compromised due to intraruminal metabolic processes, caused by rumen microbiota. Specifically, biohydrogenation reduces the concentration of unsaturated FAs reaching the duodenum, thereby limiting their availability for deposition in various tissues [17]. In addition, linseed is known to contain antinutritional factors such as linamarin, a cyanogenic glycoside. Consequently, the European Food Safety Authority proposed a maximum dietary inclusion of 5% on a dry matter (DM) basis for fattening cattle [18]. Thus, the application of heat treatments, such as extrusion, have been proposed as a strategy to mitigate rumen biohydrogenation process and potentially reduce the concentration of linseed antinutritional factors [17,19].

Although extruded linseed is highly regarded for its beneficial effects, studies in cattle have mostly focused on its use to increase PUFA concentration in milk, rather than meat [20,21,22,23]. To the best of our knowledge, there are no studies evaluating the impact of extruded linseed on young Holstein bulls’ performance, meat quality and FA profile. Therefore, the objective of this study was to investigate the effects of extruded linseed dietary supplementation during the final fattening stage on the production performance and meat quality of young Holstein bulls.

## 2. Materials and Methods

### 2.1. Animals and Dietary Treatments

Sixty-eight young Holstein bulls (382 ± 37 days) were used in the present study, which took place on a commercial dairy farm near the city of Serres in Northern Greece. All young bulls were born and raised on the farm and each animal was ear-tagged. One day prior to the start of the trial, the young bulls were individually weighed to ensure they were stratified by live weight. The young bulls were randomly assigned to one of two treatments: control (CON, *n* = 34) and extruded linseed (LS, *n* = 34). The average initial age for the CON group was 385 ± 37.1 days, while the LS group had an average initial age of 379 ± 40.1 days. Subsequently, the animals in each group were allocated to one of five pens, with four pens containing seven young bulls each and one containing six young bulls. Pens were 35 m^2^ with concrete surfaces and a feed bunk capable of serving all animals simultaneously. The diets for each group were formulated to meet maintenance and growth requirements in accordance with INRA guidelines [24] for the cattle finishing period (Table 1). The control (CON) group received a total mixed ration (TMR) consisting of maize silage (20.3%), corn grain (49.1%) and soybean meal (11.9%), whereas in the linseed (LS)-supplemented group, extruded linseed was incorporated at 5% of the diet DM, replacing an equivalent proportion of corn grain (from 49.1% to 44.1%) (Table 1). The dietary treatments were administered once every morning, using a KUHN SPW Power 22.2 mixer wagon (KUHN S.A., Saverne, France) with a capacity 22.2 m^3^. Young bulls in each pen were group fed *ad libitum*. Feed provided to each pen was recorded daily (DMI; kg/d). Orts were collected and dried overnight at 80 °C to determine their dry matter content, which was then considered in the calculation of DMI. The young bulls had unlimited access to fresh water. The experimental duration was 132 ± 19.1 days. To calculate average daily gain (ADG) and feed conversion ratio (FCR), each young bull was weighed individually early in the morning before feeding, according to Albertí et al. [25], using an electronic scale at the beginning and end of the trial, with an additional intermediate weighing conducted. Since DMI was measured on a pen basis, FCR was calculated by dividing the pen basis DMI by the mean ADG of animals in each pen; the pen served as the experimental unit.

### 2.2. Slaughtering and Meat Sampling

At the end of the study, all cattle were slaughtered at a commercial abattoir located within 20 km of the farm. Steaks were excised from both left and right sides of *Longissimus dorsi* muscle at the 13th rib. Samples were obtained from beef carcasses following the methodology outlined by Tsitsos et al. [9]. The samples were transported to the laboratory in insulated polystyrene containers maintained at ≤4 °C and stored in vacuum packaging under refrigeration (≤4 °C) for further analysis.

### 2.3. Meat Quality Assessment

On the first day of storage, meat samples were subjected to physicochemical analyses, including pH measurements, colorimetry, texture profile analysis (TPA), chemical composition, FA profile and lipid oxidation.

Meat pH was assessed with a portable pH meter (FiveGo pH meter F2, Mettler Toledo, Zaventem, Belgium). The pH meter probe was inserted into each sample non-destructively. To maintain consistency, three sequential readings were taken at the same probe entry point, and their mean value was used for the analysis. The accuracy of measurements was ensured through the process of calibration prior to each analysis using two standard pH buffer solutions, one at pH 4.00 and the other at pH 7.00.

Meat color evaluation was performed on each sample, as outlined by Tsitsos et al. [27]. A Konica Minolta CR-410 Chroma Meter (Tokyo, Japan) was used, with an aperture of 50 mm, illuminant C, and a 2° standard observer. A chroma meter was calibrated using a white reference tile with the values Y: 94.8, X: 0.3130 and y: 0.3190. Three consecutive measurements made at different points were obtained from each sample, and values for lightness (L*), redness (a*) and yellowness (b*) were recorded. Measurements were made perpendicular to muscle fibers, while ensuring that areas containing fat or connective tissue were avoided. The calculation of chroma and hue angle was performed according to the proposed formulas outlined by the American Meat Science Association (AMSA) [28], taking into consideration a* and b* mean values:

Chroma = (a*^2^ + b*^2^)^1/2^

Hue angle = arctangent (b*/a*)

The TPA was conducted using a Stable Micro Systems TA.HD plus Texture Analyzer (Godalming, UK), integrated with a flat-faced cylindrical probe (diameter: 1.27 cm). Exponent software (version 6.1.16.0) was used to operate the analyzer. For each analysis, oval-shaped meat pieces 2–3 cm in width and thickness were removed from the center of each steak for analysis. A cycle of double-compression test was applied, with the probe crushing samples perpendicularly to the muscle fibers. Test parameters included a pre-test speed of 1.00 mm/s while both test and a post-test speed were set at 5.00 mm/s, achieving a 40% deformation of the sample height in each cycle. The interval between the two compressions was set at 2.02 s. The force–time plots generated during the analysis represented the sample’s resistance to compression over time, enabling the calculation of key texture parameters such as Hardness 1, Hardness 2, Cohesiveness, Springiness, Chewiness, Gumminess and Resilience, as described by Skaperda et al. [29] and Peleg [30].

Meat chemical composition was carried out in accordance with the methodology described by Tsitsos et al. [9]. Prior to the analysis, 100 g of meat was weighed and transferred to a plastic sample container for the evaluation; this evaluation was completed using a near-infrared spectrometer (NIR, Perten DA7250, Perkin Elmer Ltd., Waltham, MA, USA) calibrated specifically for meat and meat products. The calibration process followed ISO and AOAC-approved protocols, including Soxhlet extraction for fat, drying cabinet methods for moisture, the Kjeldahl method for protein, hydroxyproline analysis for collagen, muffle furnace techniques for ash, and ICP-MS for salt determination. Measurement precision and reliability were maintained through calibration models developed by Artificial Neural Networks (ANN) and Honigs Regression™.

For the determination of meat FA profile, 100 g from each sample was homogenized. Subsequently FAs were extracted using the Soxtherm Soxhlet Extraction System following AOAC Method 991.36, detailed by Tsitsos et al. [9]. This was followed by a trans-esterification in a methanolic potassium hydroxide solution. The certified reference standard CRM 47885 Supelco 37 Component FAME Mix was used to identify the peaks of individual fatty acid methyl esters (FAMEs). The resulting FAMEs samples were then analyzed using Gas Chromatography with Flame Ionization Detection (GC-FID). Chromatographic analyses were carried out with a Shimadzu GC-2010 Plus High-End gas chromatography system equipped with an FID detector. A Supelco SP2560 100 m × 0.25 mm × 0.20 μm column was used. Helium (grade 99.999%) served as a carrier gas operating at a flow rate of 2 mL/min. The injection volume was 1 μL with a split ratio of 1:50 and the injector temperature was set at 250 °C. The detector temperature was set at 250 °C. The temperature program employed was as follows: initial oven temperature at 110 °C (7 min), increasing at 3 °C/min to 190 °C (2 min), then in the first step at 0.5 °C/min to 205 °C, in a second at 5 °C/min to 230 °C (5 min) and in a third at 5 °C/min to a final temperature of 240 °C for 5 min. The total run time was 82.67 min. FAs were identified and reported as relative percentages (%) of the total FAs detected.

To evaluate lipid oxidation induced by refrigerated storage, malondialdehyde (MDA) concentrations were measured using the method described by Ahn et al. [31]. Samples were vacuum-packed and stored in a refrigerator at 4 °C. Analyses were conducted on Days 1, 5, and 10 of storage. MDA concentrations were determined by comparing sample absorbance to a standard curve prepared with known concentrations of MDA and were expressed as mg/kg of meat.

### 2.4. Statistical Analysis

All statistical analyses were performed using IBM SPSS Statistics software (version 29.0, IBM Corporation, Armonk, NY, USA). The Shapiro–Wilk test was used to assess the normality of data distribution. An analysis of variance (ANOVA) was conducted to evaluate the effect of extruded linseed dietary supplementation on performance parameters, meat quality traits and FAs. Homogeneity of variances was assessed using Levene’s test. The treatment groups (CON and LS) were considered as fixed effects with the pen serving as the experimental unit. To account for the lack of independence among animals housed within the same pen, all individual measurements were averaged per pen prior to statistical analysis. The parameters that did not follow a normal distribution (hot carcass weight, gumminess and chewiness) were analyzed using the Mann–Whitney U test. A statistical significance level of 0.05 was set for all cases.

## 3. Results and Discussion

A potential barrier associated with extruded linseed supplementation in the diets of fattening young bulls is its relatively high cost [32]. Considering that feed represents the largest expense of feedlot farms [33] and that beef systems that use dairy breeds typically operate at low profit margins [6], extruded linseed supplementation must be carefully optimized. In contrast to previous studies evaluating both extruded [34] and non-extruded linseed [35,36], our experimental design applied extruded linseed supplementation for a shorter period, restricted to the final fattening stage, and at a lower inclusion rate. This approach was adopted primarily in light of the high cost of extruded linseed, making long-term supplementation less feasible in commercial production systems.

### 3.1. Animal Performance

Confirmation that both groups were exposed to the dietary treatment for a comparable period was made through statistical analysis (CON: 131 ± 15.6 days; LS: 132 ± 23.0 days). The effects of extruded linseed dietary supplementation on young Holstein bulls’ performance parameters are summarized in Table 2. As animals were stratified based on live weight at the beginning of the experiment there were no significant differences in the initial body weight between experimental groups (*p* > 0.05). Regarding slaughter weight, the dietary intervention did not result in significant differences between groups (*p* > 0.05). At slaughter, the average body weights of the CON and LS bulls were 626 ± 43.2 and 642 ± 34.6 kg, aged 516 ± 33.6 and 511 ± 28.4 days, respectively. In addition to slaughter weight, ADG was similar between the CON (1.46 ± 0.2 kg/day) and LS (1.49 ± 0.1 kg/day) groups (*p* > 0.05). Finally, extruded linseed supplementation was not associated with any negative effect on DMI and FCR in the present study (*p* > 0.05). The latter findings are consistent with those reported by Ragni et al. [34], who supplemented extruded linseed at the level of 35% on DM basis in the concentrated feed, with wheat straw offered ad libitum in the diet of young Podolica bulls. Similarly, in a study involving Limousin and Charolais heifers, extruded linseed supplementation at the level of 7% on a DM basis resulted in no significant effects on the aforementioned performance parameters [34]. Our results further corroborate previous studies on Holstein [35], Simmental [13] and Pirenaica bulls [25], which evaluated the effects of non-extruded dietary supplementation and reported no adverse effects on ADG, DMI and FCR. This outcome was anticipated, as diets were formulated to be isoenergetic and were offered to young bulls of the same breed under consistent environmental conditions. Moreover, in the present study, young bulls were supplemented with extruded linseed received a diet with high fat content. It is well documented that dietary fats, particularly PUFA, can disrupt rumen microbial balance, potentially leading to DMI reduction. However, in this trial, the total ether extract concentration of LS diet was formulated to remain just below the reported upper limits 6–7% for feedlot cattle [37,38]. Furthermore, the fat source used underwent a heat treatment which is known to reduce its adverse effects on rumen [19]. Notably, the ADG observed in this study was higher than expected based on the growth potential of Holstein bulls finished on concentrate-based diets, as reported in previous studies, where ADG fluctuated between 1.18 and 1.25 kg/day [36,39,40].

Extruded linseed supplementation had no significant effect on dressing percentage or hot carcass weight in young Holstein bulls (*p* > 0.05) (Table 2). In contrast to our findings, Ragni et al. [34] reported increased dressing percentage, without differences in carcass yield, in Podolica bulls supplemented with extruded linseed at the level of 350 g/kg DM of concentrated feed. According to the authors, this outcome was attributed to a reduction in perirenal fat in the treated animals. Additionally, the inclusion level of extruded linseed was substantially higher in the study by Ragni et al. [34] compared to ours. It should be noted that perirenal fat was not measured in the present study. Nevertheless, our findings align with the majority of previously published studies that investigated non-extruded linseed supplementation in fattening bulls [35,36,41]. However, Albertí et al. [25] reported a reduced dressing percentage in Pirenaica bulls fed 5% non-extruded linseed, attributing this outcome to the mucilage content of the seed. Mucilage is a polysaccharide known for its ability to absorb water and increase gut fill, which could lower dressing percentage. By comparison, in our study, linseed was thermally processed, which likely reduced the functional properties of mucilage [42]. Overall, the mean dressing percentages observed in our study were relatively low, yet they remained within the typical range for dairy breeds [7,39,43,44].

### 3.2. Meat Quality Characteristics

The effect of extruded linseed dietary supplementation on meat pH is reported in Table 3. Notably, meat from LS bulls showed significantly lower pH values compared to the CON (*p* = 0.011), suggesting improved meat quality. Meat pH is an important quality indicator, as it influences meat shelf life, color, tenderness and flavor. Several factors such as age, gender, diet, farm management practices and handling throughout the slaughter process are responsible for the meat pH value [45]. In particular, muscle glycogen reserves during the pre-slaughter phase serve as the metabolic substrate for post-mortem lactate production through glycolysis, which leads to a decline in pH [46,47,48]. It is generally accepted that pH values exceeding 5.8 are associated with a deterioration in meat quality [9]. In the present study, meat pH values for both CON (5.7) and LS (5.6) groups were within the desirable range. Although a significant reduction in meat pH was observed in LS group, our findings differ from those of Ragni et al. [34], the only study evaluating the effects of extruded linseed in fattening bulls, which reported no significant alteration of meat pH. In addition, the results of studies evaluating non-extruded linseed are inconsistent. Our findings align with Kaić et al. [13] who reported a correlation between lower meat pH and non-extruded linseed supplementation in Simmental bulls. According to the authors, this outcome may be attributed to linseed’s potential interference with the speed of post-mortem glycolysis. However, other studies have reported no significant effect of whole non-extruded linseed supplementation on the meat pH of fattening bulls [25,36].

Consumers’ purchasing decisions are highly correlated with the visual appearance of beef. In the present study, the dietary intervention had no effect in terms of meat color parameters (*p* > 0.05) (Table 3). In contrast to our results, extruded linseed supplementation led to the increased lightness (L*) of the Longissimus lumborum muscle of Podolica bulls, although the remaining measured color parameters did not differ significantly [34]. However, our results are consistent with previous studies that reported no significant effects of non-extruded linseed on meat color parameters [13,36,41]. Consumers generally prefer bright, cherry-red steaks [49], making meat redness (a*) a key indicator of beef acceptability [50,51]. Meat redness is affected by diet, environmental factors and slaughter conditions, which influence the oxidative status of myoglobin in muscles [52]. Specifically, high pH values favor the conversion of myoglobin to metmyoglobin, leading to brown and less desirable color shades. Conversely, low pH values promote the formation of oxymyoglobin, which is associated with an appealing bright red color [50]. Overall, the redness values of steaks in our experiment were above the threshold of 14.5 proposed by Holman et al. [51], which is considered indicative of consumer acceptability.

Extruded linseed dietary supplementation had no statistically significant effect on meat protein or ash concentration (*p* > 0.05) (Table 3). Moreover, intramuscular fat did not significantly differ across treatments (*p* > 0.05), indicating that dietary intervention had no influence on meat chemical composition. Among the available literature assessing the effects of extruded and non-extruded linseed on the chemical composition of meat, results remain inconsistent. High-level supplementation of extruded linseed (350 g/kg DM of concentrated feed) was associated with increased muscle fat but decreased moisture and protein content [34]. Furthermore, Kaić et al. [13] observed a significant increase in muscle protein and ash content with no effect on fat in Simmental bulls supplemented with 160 g of non-extruded linseed, indicating a potential role in enhancing their deposition in muscles. By contrast, extruded linseed supplementation had no effect on the chemical composition of meat from crossbred steers [53] or Limousin heifers [54]. The findings from the aforementioned studies align with Albertí et al. [35,25], who reported no significant effects on muscle protein, ash and fat content, following non-extruded linseed supplementation at 10% and 5% on a DM basis, respectively. Muscle protein and ash content show negligible variation among cattle during the finishing period, as growth phase is the main factor influencing their concentration [55]. In contrast, beef intramuscular fat shows greater variability due to the influence of multiple factors, such as genetics, sex, growth stage, farm management and nutrition [56]. Among these, dietary energy is considered the most critical nutritional factor influencing its concentration [55,56]. In the present study the energy content of diets was very similar; therefore, they were considered isoenergetic, which may account for the absence of difference in meat fat content. Overall, the meat chemical composition observed in our study aligns with reported values for meat from Holstein bulls weighing over 500 kg, with protein ranging from 20 to 22%, moisture content between 70 and 75%, fat around 2–3% and ash between 1 and 2% [9,57,58].

Beef tenderness is an essential quality feature that influences consumer preferences, as it reflects the ease of meat mastication [59]. This attribute varies considerably due to biological and environmental factors [60]. In our study, meat tenderness was assessed using an instrumental method (TPA) (Table 3), which is reported to offer strong accuracy compared to sensory analysis [61]. According to our findings, no statistically significant differences were found in beef Hardness 1 and 2 between the two groups (*p* > 0.05). To the best of our knowledge, this is the first study to investigate the effect of extruded linseed dietary supplementation on beef tenderness using the TPA method. This method is reported to exhibit superior accuracy in predicting meat hardness [62]. However, Warner-Bratzler shear force method was used by several authors who reported no significant effects of non-extruded linseed on beef tenderness. For instance, Ragni et al. [34] examined the effect of extruded linseed on the tenderness of Longissimus lumborum muscle in Podolica bulls and observed similar results between supplemented and control groups. Likewise, Razminowicz et al. [54] reported no effect of extruded linseed dietary supplementation on the Longissimus dorsi muscle tenderness of crossbred steers. Finally, Corazzin et al. [36] evaluated the effect of non-extruded linseed supplementation on beef tenderness and found no significant differences in the Longissimus thoracis muscle of Simmental and Holstein bulls. Overall, beef in our study is considered tender, as the values for both Hardness 1 (CON: 1659.4 g = 16.3 N; LS 1630.1 g = 16.0 N) and 2 (CON: 1298.7 g = 12.7 N; LS: 1344.5 g = 13.2 N) fell below the threshold of 31.89 N proposed by Ricardo-Rodrigues et al. [59] to define tender beef.

The physicochemical parameters evaluated in the present study, such as meat pH, color and texture, are closely related to visible and sensory attributes that influence consumer acceptance [63]. Although water holding capacity and water activity were not directly assessed, they are known to affect meat color, particularly L*, as well as texture parameters such as cohesiveness [64,65]. In our study, no significant differences were observed between the CON and LS groups regarding L* or texture parameters. Given that all samples were collected and analyzed under identical, standardized conditions, the absence of WHC and aw measurements is not considered to compromise the reliability of the results. Nonetheless, incorporating such analyses in future research would allow for a more comprehensive assessment of meat quality and consumer acceptance traits.

Beef FA composition is influenced by several factors, including age, breed and nutrition [66]. Due to low n-3 intake in Western diets, much attention has been given to increasing n-3 intake via animal products and to raising consumer awareness of their nutritional value [67,68]. As outlined in the introduction, thermally processed linseed was selected in this study to reduce rumen biohydrogenation of dietary FAs, aiming to increase α-linolenic acid deposition in muscles. Table 4 displays the effects of the dietary intervention on the FA profile of the *Longissimus dorsi* muscle, with values expressed as % of total FAs. The most abundant FAs were oleic (C18:1 n-9 cis, CON: 37.4%, LS: 37.3%), palmitic (C16:0, CON: 28.9%, LS: 29.3%) and stearic acid (C18:0, CON: 19%, LS: 18.5%), with no significant differences observed between the groups (*p* > 0.05). The only SFA that showed a mild but significant reduction in LS group compared to CON was C20:0. A similar finding was reported by Rangi et al. [34] in bulls fed extruded linseed, as well as in trials where convectional linseed was used [13,36]. While the exact mechanism in not fully understood, it is possible that the reduction in C20:0 reflects an effect on SFA elongation, as C20:0 is a known elongation product of C18:0. Accordingly, no differences were reported in the overall SFA and MUFA concentrations among the bulls. In contrast, the inclusion of 5% extruded linseed in the diet resulted in a significant 1.72-fold increase in α-linolenic acid (C18:3 n-3) concentration in the muscle of Holstein bulls compared to CON group (*p* < 0.001). Likewise, total n-3 FAs were higher in LS group (*p* < 0.001). These results are consistent with findings by Ragni et al. [34] who reported a 2-fold increase in α-linolenic acid when extruded linseed was supplemented in the diet of Podolica young bulls. Additionally, studies by Corazzin et al. [36], Kaić et al. [13] and Albertí et al. [25] reported 1.38, 2.00 and 2.55-fold increases, respectively, in α-linolenic acid content when non-extruded linseed was included in the diets of fattening bulls. α-linolenic acid appears to play an important role in human health, as widely documented in the literature [69,70]. It has been linked not only to the protection of the cardiovascular system but also to positive effects on the immune system [69,70]. For this reason, the increase in a-linolenic acid observed in the beef from LS bulls is considered highly beneficial and adds nutritional value to the final product.

**Figure 1:**
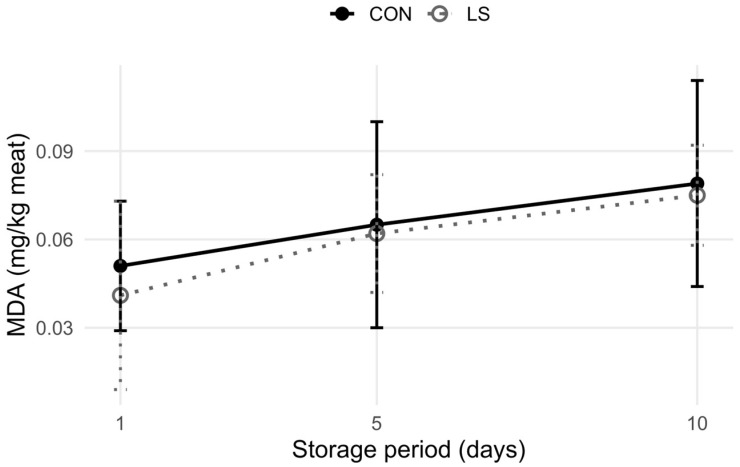
Lipid oxidation, expressed as MDA (mg/kg of meat), in Longissimus dorsi muscle of young Holstein bulls fed a control (CON) and an extruded linseed-supplemented (LS) diet, measured after 1, 5 and 10 days of refrigerated storage. No significant differences were observed between treatments at any time point (*p* > 0.05 for time points).

MDA in meat, a product of secondary lipid oxidation, is commonly used as a marker of oxidative stability in meat products [71]. Lipid oxidation is a major factor contributing to the spoilage of refrigerated meat, significantly deteriorating its quality and diminishing consumer acceptance. The concentration of MDA in muscle from bulls supplemented with extruded linseed did not differ significantly from that of the control (CON) bulls (*p* > 0.05) (Figure 1). This finding remained consistent across all examined time points (days 1, 5 and 10) during refrigerated storage. Furthermore, MDA values remained below the threshold of 2 mg/kg of meat for both groups, which is widely recognized as the level at which rancidity becomes perceptible to consumers [72]. These results align with those reported by Ragni et al. [34], who found that extruded linseed supplementation in Podolica bulls did not lead to elevated MDA concentrations in meat. In contrast, increased lipid oxidation was reported by Kaić et al. [13] following non-extruded linseed supplementation in bulls. Overall, diets high in polyunsaturated fatty acids (PUFAs) have been associated with reduced oxidative stability in meat [73]. Although n-3 FAs, which are highly prone to oxidation, were increased in our study, MDA levels in the LS group were not elevated. Proper meat storage conditions play a key role in minimizing and delaying lipid oxidation [74]. In our study, the samples were vacuum-packed and stored at 4 °C. The storage conditions may have contributed to the absence of lipid oxidation increase in LS beef, along with the fact that total PUFA levels remained similar between the groups despite n-3 FAs increase.

## 4. Conclusions

The results of the present study indicate that the inclusion of 5% extruded linseed in the diet of young Holstein bulls, during the final fattening stage, had no adverse effect on performance parameters. Most of the examined meat quality traits were not significantly affected by extruded linseed supplementation. However, a significant reduction in meat pH was observed, suggesting improved post-mortem glycolysis and potential benefits for meat quality. Notably, α-linolenic acid, the primary n-3 FA in beef, showed a 1.72-fold increase without compromising lipid oxidation stability. Such feeding strategies can support the utilization of young Holstein bulls from domestic dairy farms and also enhance the nutritional value of beef to meet consumer expectations and market trends.

## Figures and Tables

**Table 1:** Ingredients and nutritional composition of the experimental diets fed to young Holstein bulls.

Item	CON	LS
Ingredient (% DM basis)		
Corn silage	20.3	20.3
Wheat straw	10.2	10.1
Corn grain, ground	49.1	44.1
Soybean meal	11.9	11.9
Cottonseed	6.2	6.2
Whole linseed	0.0	5.1
Sodium bicarbonate	0.3	0.3
Sodium chloride	0.3	0.3
Vitamin mineral premix	1.6	1.6
**Chemical analysis**		
Crude protein, % of DM	13.94	14.65
Crude fiber, % of DM	12.12	12.49
Ether extract, % of DM	4.19	5.88
Starch, % of DM	39.70	36.52
Neutral detergent fiber, % of DM	26.97	27.50
Acid detergent fiber, % of DM	14.10	14.63
UFV ^1^, kg DM	1.05	1.07
**Fatty acid composition ^2^** **(% total fatty acids)**		
C16:0	9.26	7.36
C18:0	2.21	2.03
C18:1 n-9	9.60	8.89
C18:2 n-6	20.52	16.34
C18:3 n-3	0.12	6.22

^1^ Net energy content for maintenance and meat production. ^2^ Fatty acid profile of the diets were calculated based on matrix values provided in the Premier Nutrition Atlas [26] (Premier Nutrition, Rugeley, UK).

**Table 2:** Performance and carcass characteristics of young Holstein bulls fed a control (CON) and an extruded linseed-supplemented (LS) diet.

	Treatments ^1^			
	CON	LS	SEM ^2^	*p*-Value
Initial BW, kg	436.3	444.2	11.260	0.749
Final BW, kg	625.8	641.6	11.964	0.548
Average daily gain, kg	1.46	1.49	0.043	0.740
Average daily DMI ^3^, kg/Days	14.0	13.6	0.476	0.684
FCR ^4^	10.2	9.3	0.560	0.432
Hot carcass weight ^5^, kg	312.4	314.9	4.60, 6.40	0.421
Dressing percentage (%)	50.8	51.0	0.155	0.593

^1^ CON = control group; LS = extruded linseed-supplemented group. ^2^ SEM = Standard error of the means. ^3^ DMI = Dry Matter Intake. ^4^ FCR = Feed conversion ratio. ^5^ Instead of means and SEM, the medians and average ranks of the Mann–Whitney (Wilcoxon) test are reported.

**Table 3:** Meat quality traits of the Longissimus dorsi muscle from young Holstein bulls fed a control (CON) and an extruded linseed-supplemented (LS) diet.

	Treatments ^1^			
	CON	LS	SEM ^2^	*p*-Value
pH	5.7	5.6	0.014	0.011
Lightness-L*	35.4	35.9	0.685	0.774
Redness-a*	16.8	17.8	0.400	0.224
Yellowness-b*	8.6	8.9	0.476	0.310
Chroma	19.0	20.0	0.459	0.297
Hue angle°	0.46	0.46	0.021	0.800
Hardness1 (g)	1659.4	1630.1	198.892	0.946
Hardness2 (g)	1298.7	1344.5	162.236	0.897
Adhesiveness (g)	−7.5	−7.5	0.912	0.985
Springiness	0.7	0.7	0.016	0.352
Cohesiveness	0.5	0.6	0.014	0.311
Gumminess ^3^ (g)	695.3	966.2	5.0, 6.0	0.690
Chewiness ^3^ (g)	519.0	695.4	4.6, 6.4	0.421
Resilience	0.3	0.3	0.015	0.515
Moisture (%)	74.0	74.8	0.372	0.321
Protein (%)	22.6	21.7	0.349	0.212
Fat (%)	3.2	3.2	0.204	0.920
Collagen (%)	1.7	1.7	0.057	0.780
Salt (%)	1.0	0.9	0.087	0.848
Ash (%)	1.1	1.1	0.091	0.929

^1^ CON = Control group; LS = Extruded linseed-supplemented group. ^2^ SEM = Standard error of the means. ^3^ Instead of means and SEM, the medians and average ranks of the Mann–Whitney (Wilcoxon) test are reported.

**Table 4:** Fatty acid profile (%) of the Longissimus dorsi muscle of young Holstein bulls fed a control (CON) and an extruded linseed-supplemented (LS) diet.

	Treatments ^2^			
Fatty Acid ^1^	CON	LS	SEM ^3^	*p*-Value
C14:0	4.352	4.324	0.053	0.810
C14:1	1.110	1.138	0.021	0.534
C15:0	0.438	0.456	0.008	0.260
C16:0	28.914	29.320	0.127	0.112
C16:1	3.622	3.716	0.114	0.705
C17:0	0.882	0.906	0.016	0.494
C17:1	0.496	0.472	0.010	0.235
C18:0	19.022	18.472	0.276	0.349
C18:1 n-9 trans	0.174	0.166	0.009	0.697
C18:1 n-9 cis	37.404	37.276	0.305	0.848
C18:2 n-6 trans	0.340	0.292	0.017	0.179
C18:2 n-6 cis	2.290	2.474	0.068	0.191
C18:3 n-3	0.210	0.362	0.025	<0.001
C20:0	0.086	0.072	0.003	0.002
C20:1 n-9 cis	0.118	0.098	0.006	0.066
C21:0	0.140	0.100	0.001	0.141
Σn-6 ^4^	2.658	2.730	0.078	0.670
Σn-3 ^5^	0.230	0.390	0.027	<0.001
ΣMUFA ^6^	43.462	42.600	0.495	0.416
ΣPUFA ^7^	2.898	3.132	0.091	0.214
ΣSFA ^8^	53.210	54.238	0.421	0.244

^1^ All values are expressed as relative percentages (%) of total fatty acids. ^2^ CON = control group; LS = extruded linseed-supplemented group. ^3^ SEM = Standard error of the means. ^4^ Σn-6 = Total concentration of omega-6 polyunsaturated fatty acids. ^5^ Σn-3 = Total concentration of omega-3 polyunsaturated fatty acids. ^6^ ΣMUFA = Total concentration of monosaturated fatty acids. ^7^ ΣPUFA = Total concentration of polysaturated fatty acids. ^8^ ΣSFA = Total concentration of saturated fatty acids.

## Data Availability

The dataset is available upon request from the authors.
